# An archaeal Cas3 protein facilitates rapid recovery from DNA damage

**DOI:** 10.1093/femsml/uqad007

**Published:** 2023-02-09

**Authors:** Guy Miezner, Israela Turgeman-Grott, Kelly M Zatopek, Andrew F Gardner, Leah Reshef, Deepak K Choudhary, Martina Alstetter, Thorsten Allers, Anita Marchfelder, Uri Gophna

**Affiliations:** The Shmunis School of Biomedicine and Cancer Research, George S. Wise Faculty of Life Sciences, Tel Aviv University, Tel Aviv 69978-01, Israel; The Shmunis School of Biomedicine and Cancer Research, George S. Wise Faculty of Life Sciences, Tel Aviv University, Tel Aviv 69978-01, Israel; New England Biolabs, Inc., 240 County Road, Ipswich, MA 01938, USA; New England Biolabs, Inc., 240 County Road, Ipswich, MA 01938, USA; The Shmunis School of Biomedicine and Cancer Research, George S. Wise Faculty of Life Sciences, Tel Aviv University, Tel Aviv 69978-01, Israel; The Shmunis School of Biomedicine and Cancer Research, George S. Wise Faculty of Life Sciences, Tel Aviv University, Tel Aviv 69978-01, Israel; Department of Biology II, Ulm University, 89069 Ulm, Germany; School of Life Sciences, University of Nottingham, Nottingham NG7 2UH, UK; Department of Biology II, Ulm University, 89069 Ulm, Germany; The Shmunis School of Biomedicine and Cancer Research, George S. Wise Faculty of Life Sciences, Tel Aviv University, Tel Aviv 69978-01, Israel

**Keywords:** Cas3, DNA-repair, CRISPR, recombination, haloferax, archaea

## Abstract

CRISPR-Cas systems provide heritable acquired immunity against viruses to archaea and bacteria. Cas3 is a CRISPR-associated protein that is common to all Type I systems, possesses both nuclease and helicase activities, and is responsible for degradation of invading DNA. Involvement of Cas3 in DNA repair had been suggested in the past, but then set aside when the role of CRISPR-Cas as an adaptive immune system was realized. Here we show that in the model archaeon *Haloferax volcanii* a *cas3* deletion mutant exhibits increased resistance to DNA damaging agents compared with the wild-type strain, but its ability to recover quickly from such damage is reduced. Analysis of *cas3* point mutants revealed that the helicase domain of the protein is responsible for the DNA damage sensitivity phenotype. Epistasis analysis indicated that *cas3* operates with *mre11* and *rad50* in restraining the homologous recombination pathway of DNA repair. Mutants deleted for Cas3 or deficient in its helicase activity showed higher rates of homologous recombination, as measured in pop-in assays using non-replicating plasmids. These results demonstrate that Cas proteins act in DNA repair, in addition to their role in defense against selfish elements and are an integral part of the cellular response to DNA damage.

## Teaser

**One sentence summary:** Cas3 helicase activity is required for rapid recovery from DNA damage and associated with microhomology-mediated end-joining in the model *Haloferax volcanii*.

## Introduction

*Haloferax volcanii* is a halophilic archaeon and an important archaeal model species, with many genetic tools and different “omics”-approaches available. Work on DNA damage repair in *H. volcanii* has shown that DNA double-strand breaks can be repaired either by homologous recombination (HR) or by a process known as microhomology-mediated end joining (MMEJ) (Marshall and Santangelo 2020; Pérez-Arnaiz et al. 2020). MMEJ relies on the resection of exposed DNA ends by exonucleases, which expose short stretches of single-stranded DNA that are homologous to one another and enable the two strands to anneal (White and Allers 2018). Unlike MMEJ, which inevitably results in deletion of a DNA fragment due to the resection and reannealing process, repair by HR generally keeps the genetic information intact and is usually error-free. Nonetheless, in *H. volcanii*, HR is restrained by the presence of two proteins, Mre11 and Rad50, that act to prevent HR and thereby allowing MMEJ to act as the primary double-strand break repair pathway (Delmas et al. 2009). These two proteins, which are highly conserved in archaea and eukaryotes, are also required for DNA compaction following DNA damage (Delmas, Duggin, and Allers 2013). When the genes for Mre11 and Rad50 are deleted, HR becomes the dominant mode of repair in *H. volcanii*, which results in slower recovery from DNA damage but also improved cell survival rates (Delmas et al. 2009). Thus, there appears to be a trade-off in *H. volcanii* between repair speed (using rapid but inaccurate MMEJ) and survival (using slow but accurate HR).

CRISPR-Cas systems provide acquired and heritable immunity to archaea and bacteria against selfish mobile elements, especially viruses, by degrading the nucleic acids of invading elements that match the DNA-based immune memory stored in CRISPR arrays. Cas3 is a CRISPR-associated protein that is common to all Type I systems. Cas3 possesses both nuclease and helicase activities (Makarova et al. 2002, 2006; Sinkunas et al. 2011; Gong et al. 2014) and after being recruited by a multiprotein complex bound to invader-targeting crRNA, is responsible for degradation of invading DNA (Maier, Dyall-Smith, and Marchfelder 2015; Hille et al. 2018). Involvement of Cas3 in DNA repair had been suggested in the past (Makarova et al. 2002), but this proposal was set aside when the canonical role of CRISPR-Cas as an adaptive immune system was realized. Previous functionality observation of the CRISPR-Cas system in DNA repair in *Escherichia coli* (Babu et al. 2011), encouraged us to reexamine the earlier ideas of Cas3 and its specific involvement in DNA repair. Here we use a genetic approach to unravel the interaction of Cas3 with the DNA repair machinery of *H. volcanii*, and how it affects HR in this species.

## Results

### A *Δcas3 H. volcanii* mutant shows higher resistance to DNA damaging agents

**Figure 1.**
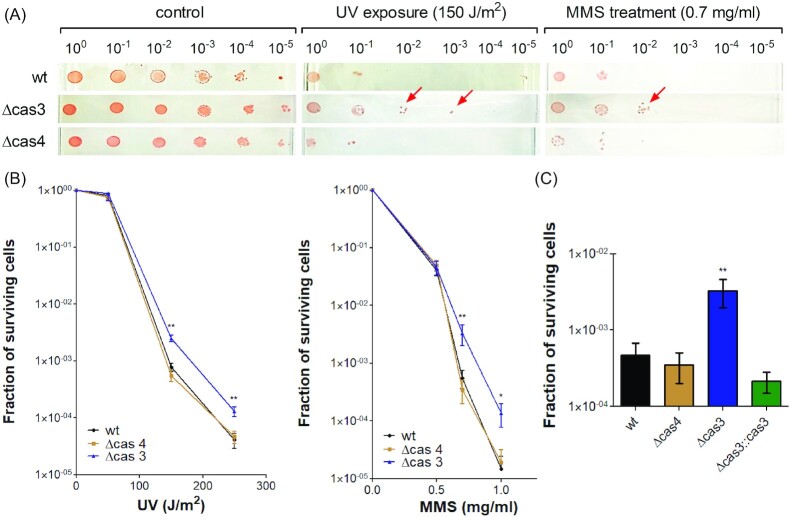
*Δcas3* mutant shows higher resistance to DNA damage. WT (H26), *Δcas3* (UG610), and *Δcas4* (UG611) cultures were exposed to DNA damage by UV or MMS treatment and plated on Hv-YPC agar. (A) Photographs were taken after 5–10 days (red arrows mark the surviving colonies). (B) Surviving colonies following different UV and MMS doses were enumerated, and the fraction of surviving cells for each strain was calculated. The mean and SE of at least six experiments are shown. (C) Fraction of surviving cells following 0.7 mg/ml MMS treatment. The mean and SE of nine experiments are shown. * = *P*-value <.05; ** = *P*-value <.01, Mann–Whitney test, compared to WT.

To test whether *cas* genes play a role in DNA repair in *H. volcanii*, we constructed two *H. volcanii* mutants. The first was deleted for all *cas* genes, including both CRISPR loci from pHV4 (*Δcas genes*) and the second had a deletion of just the *cas3* gene (*cas3-*KO) (Supplementary Fig. S1). We examined the sensitivity of both mutants to DNA damage, utilizing ultraviolet (UV-C) exposure as a DNA damaging agent (Delmas et al. 2009). Notably, the *cas3-*KO mutant showed significantly higher resistance to UV damage compared to both the wild-type (WT) strain and the *Δcas genes* mutant, as observed by colony formation at higher dilutions (Supplementary Fig. S2). Since the *cas3*-KO mutant included the deletion of the entire *cas3* gene, which resulted in deletion of a potential *cas4* promoter, we constructed new separate *Δcas3* and *Δcas4* mutants (Supplementary Fig. S1), to determine which of these two *cas* genes is responsible for the UV-resistance phenotype. Those new mutants were then exposed to DNA damaging agents, both UV-C and the DNA-alkylating mutagen methyl methanesulphonate (MMS) (Delmas et al. 2009). In both cases, the resistance of the *Δcas3* mutant to the DNA damaging agents was significantly higher than the WT strain and the *Δcas4* mutant, as observed by colony formation of the *Δcas3* mutant in the higher dilution series (Fig. 1). To confirm that DNA damage resistance was related to the deletion of the *cas3* gene, we conducted a complementation assay, transforming an expression plasmid (pTA927) with the *cas3* gene under the tryptophan-inducible promoter p.*tnaA* to the *Δcas3* strain. As expected, the MMS sensitivity of *Δcas3* mutant was restored to the WT levels by the induced expression of the *cas3* gene from that expression plasmid (Fig. 1 C). These results suggested a possible involvement of Cas3 in DNA damage response in *H. volcanii*. Moreover, the contrast between the resistance of the *Δcas3* mutant and the sensitivity of the *Δcas genes* mutant to DNA damaging agents, suggests that different *cas* genes might affect the susceptibility to DNA damage differently and in opposing directions in *H. volcanii*. Indeed, Cas1 contributes to DNA repair in *E. coli* (Babu et al. 2011), has been recently shown to contribute to DNA repair in *H. volcanii*, and its deletion alongside the other *cas* genes could increase sensitivity to DNA damage (Wörtz et al. 2022).

### *Cas3* promotes rapid recovery from DNA damage

In the absence of foreign invading elements, the maintenance of CRISPR-Cas systems is assumed to be a burden on cells expressing them, due to the energetic cost of constitutive expression of the interference machinery, including the *cas3* gene that is always expressed in *H. volcanii* (Brendel et al. 2014). However, the *Δcas3* mutants show no difference in growth rate compared to the WT strain under optimal conditions (Supplementary Fig. S3 A), a result which was also corroborated by direct head-to-head competition in liquid medium (Supplementary Fig. S3 B). This suggests that *cas3* does not have a significant impact on the cell’s fitness in the absence of infection by viruses or other exogenous stress.

**Figure 2.**
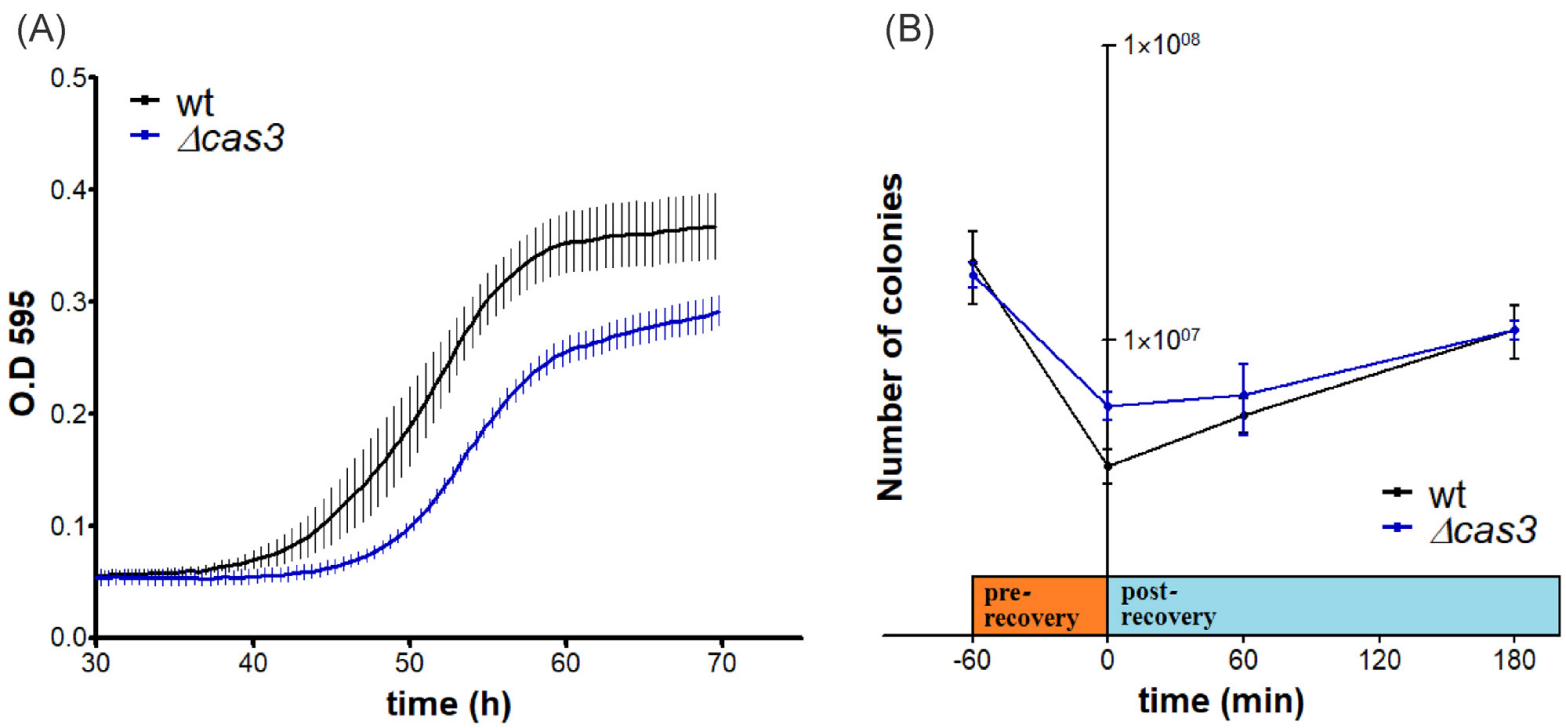
Cas3 expression promotes rapid recovery from DNA damage. (A) Growth curves of WT (H26) and Δcas3 (UG610) following treatment with 0.7 mg/ml of MMS. The mean and SE of three biological replicates are shown for each time point. (B) Recovery assay. Colonies were enumerated after 5–10 days of incubation at different time points, before and after 1 h of incubation with 0.1 mg/ml MMS (prerecovery), and after 1 to 3 h of MMS removal (post-recovery). The mean and SE of three experiments are shown.

Nonetheless, when we exposed the *H. volcanii* cultures to MMS, the *Δcas3* mutant showed significant delay in growth compared with the WT strain (Fig. 2 A). A similar result was observed in DNA damage recovery assay (Fig. 2 B), where after the first hour of MMS treatment, the mutagen was washed off and removed, and the cultures were left to recover under the same conditions for different time periods.

**Figure 3.**
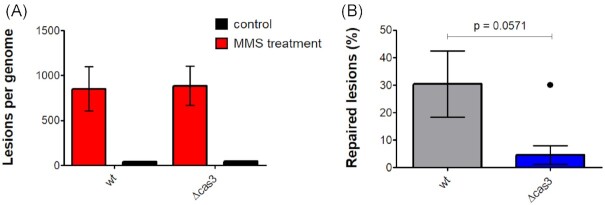
Cas3 promotes rapid repair of MMS lesions. (A) MMS lesions quantification following a 1-h MMS (0.1 mg/ml) treatment. The mean and SE of four biological replicates are shown. (B) Percentage of repair after 1-h MMS (0.1 mg/ml) treatment and 1 h of recovery time. The mean and SE of four biological replicates are shown. *P* = 0.0571 Mann–Whitney test, compared to WT.

MMS predominantly creates methylated DNA lesions such as 7-methylguanine (7meG) and 3-methlyladenine (3meA), causing base mispairing and replication blocks (Beranek 1990). DNA damage caused by alkylating agents is primarily repaired by the base excision repair (BER) pathway or by DNA alkyltransferases (Lindahl and Wood 1999), but the sensitivity of cells to MMS also increases significantly when other DNA repair pathways are compromised (Lundin et al. 2005; Delmas et al. 2009). For better understanding of the recovery process and the repair of the MMS lesions, we used RAre DAmage and Repair sequencing (RADAR-seq) (Zatopek et al. 2019) on DNA extractions from WT strain and *Δcas3* mutant to quantitate 7meG and 3meA levels immediately after MMS treatment and post recovery. This method uses a DNA glycosylase in combination with Endonuclease IV to generate nicks at damaged DNA sites and then a DNA polymerase and a ligase that convert those nicks to modified bases that are detectable by PacBio sequencing (see Materials and methods section). Both the WT and the *Δcas3* mutant appear to possess a relatively similar number of lesions, with an average of 850–880 (respectively) lesions per genome after 1 h of MMS treatment (Fig. 3 A). On the other hand, the results after the recovery time showed a higher repair efficiency in the WT strain with about 30% of repair compared to 5% of repair in the *Δcas3* mutant (Fig. 3 B). Importantly, no particular hotspots of lesions could be observed in either mutant or WT, not even near the locus that partially matches one of the *H. volcanii* spacers (Fischer et al. 2012), indicating that there is no underlying site-specific CRISPR targeting involved (Supplementary Fig. S5).

Taken together, these results show that while the amount of damage caused to both strains is comparable, after recovery there is an advantage to the WT strain, containing the functional Cas3 protein, which repairs lesions faster.

### *Cas3* helicase activity is responsible for DNA damage sensitivity

Since the *H. volcanii* Cas3 protein is predicted to have both helicase and nuclease domains, as seen in well-studied bacterial homologs (Makarova et al. 2002, 2006; Haft et al. 2005; van der Oost et al. 2009), our next goal was to distinguish between these two activities to clarify which domain is responsible for sensitivity to DNA damage. For that end, we used two expression vectors (pTA927) that carried mutated *H. volcanii cas3* genes under the p.*tnaA* promoter. One of the cloned genes was defective in its nuclease domain (encoding a nuclease-dead Cas3), with the HD63-64AA double mutation that has been shown to abolish the Cas3 nuclease activity (Beloglazova et al. 2011; Howard et al. 2011; Mulepati and Bailey 2011; Sinkunas et al. 2011; Westra et al. 2012). The other plasmid encoded a Cas3 protein with a defect in its helicase domain (helicase-dead Cas3, D444A), that has been shown to be important for Cas3 helicase activity (Sinkunas et al. 2011; Westra et al. 2012). These two plasmids were transformed separately into the *Δcas3* mutant background and tested using the DNA damage assay based on MMS treatment. Since the plasmid-encoded *cas3* mutant genes were under the tryptophan induced p.*tnaA* promoter, we tested growth without tryptophan addition and with 2 mM tryptophan added.

**Figure 4.**
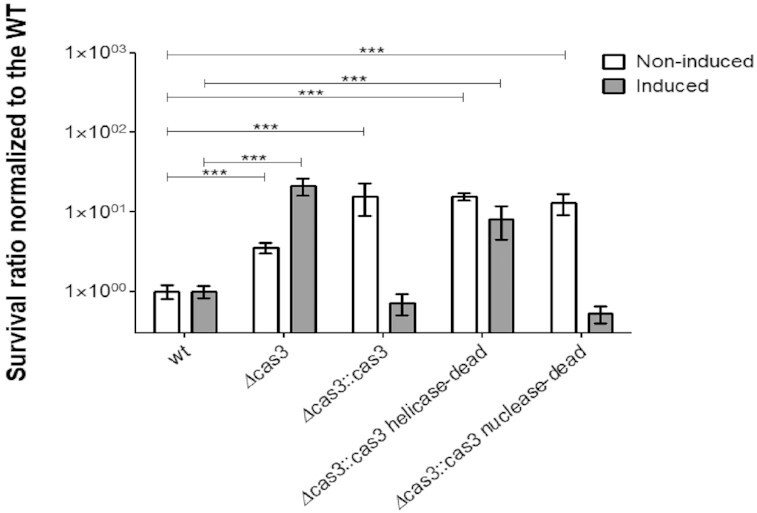
Cas3 helicase activity is responsible for DNA damage sensitivity. WT (H26), *Δcas3* (UG610), *Δcas3:: cas3* (UG617), *Δcas3:: cas3* helicase-dead (UG618), and *Δcas3:: cas3* nuclease-dead (UG645) were grown in Hv-YPC (containing 2 mM tryptophan for promoter activation) or in Hv-CA (noninduced medium) and exposed to DNA damage by incubation with 0.7 mg/ml MMS and plated on Hv-YPC. Surviving colonies were enumerated after 5–10 days of incubation and fraction of surviving cells for each mutant was normalized to the WT strain. In each case, the mean and SE of nine experiments are shown. *** = *P*-value <.001, Mann–Whitney test, compared to WT.

Under the noninducing conditions, all the four mutants (*Δcas3, Δcas3:: cas3, Δcas3:: cas3 H.D*, and *Δcas3:: cas3 N.D*) showed increased cell survival in comparison to the WT strain, following the MMS treatment. (Fig. 4). This indicates that none of the exogenous *cas3* genes were induced (or that their expression was minor due to a promoter leakage), as expected, and the resistance phenotype they all shared was due to their joint *Δcas3* background. In contrast, in the presence of tryptophan, only the *Δcas3* and the *Δcas3:: cas3 H.D* (helicase dead) mutants were still more resistant to MMS, while the *Δcas3:: cas3* and *Δcas3:: cas3 N.D* (nuclease dead) strains were more sensitive to the treatment (Fig. 4). The noncomplemented *Δcas3* strain benefited from the addition of tryptophan to the medium in terms of its survival phenotype. These results suggest that the Cas3 helicase activity mediates the sensitivity to DNA damaging agents in *H. volcanii*, while Cas3 nuclease activity has a minor role, if any, in the response to DNA damage.

### *Cas3* participates in the Mre11-Rad50 DNA repair pathway

Double strand breaks (DSBs) are considered to be one of the most destructive forms of DNA damage. Such a break, in both strands of the DNA, may be subjected to illegitimate recombination or ligation if left unrepaired, and these processes can lead to chromosomal rearrangements. Both HR and MMEJ were previously shown to be DSB repair pathways in *H. volcanii*. It is well known that HR is highly accurate and error-free with a higher energetic cost, while MMEJ is a faster, but error-prone process (Blackwood et al. 2013; Marshall and Santangelo 2020; Pérez-Arnaiz et al. 2020). Correct regulation of both error-prone and HR pathways is required for optimal DNA repair, and it appears that in *H. volcanii* the Mre11-Rad50 complex has a significant impact in committing to a specific pathway, by restraining the HR pathway and thereby allowing MMEJ to act as the primary pathway of DSB repair (Delmas et al. 2009). Interestingly, it was shown that *mre11-rad50* mutants were much more resistant to DNA damaging agents, but their recovery from the damage was slower (Delmas et al. 2009), similar to what we observed with the *Δcas3* mutant.

**Figure 5.**
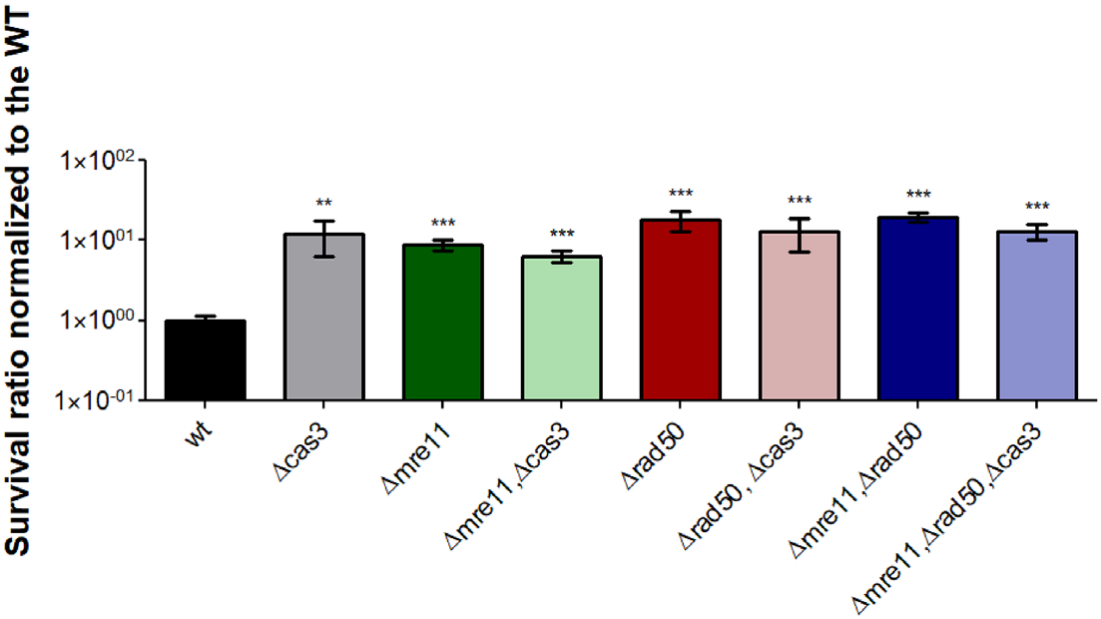
*Δcas3* shows an epistatic (nonadditive) effect in the background of *Δmre11- Δrad50*. WT (H115), *Δmre11* (H203), *Δmre11Δcas3* (UG669), *Δrad50* (H202), *Δrad50Δcas3* (UG670), *Δmre11Δrad50* (H204), and *Δmre11Δrad50Δcas3* (UG671) cells were exposed to DNA damage by 0.7 mg/ml MMS treatment and plated on Hv-YPC. Surviving colonies were counted after 5–10 days of incubation and the fraction of survival of each mutant was normalized to the WT strain. In each case, the mean and SE of nine experiments are shown. *** = *P*-value <.001, Mann–Whitney test, compared to WT.

Those observations encouraged us to examine the resistance to MMS of a mutant that has all three genes deleted: *cas3, mre11*, and *rad50*. Such an experiment might result in 2 different outcomes: the resistance effect might be enhanced and indicate that *cas3* and *mre11-rad50* operate in two different pathways (synergistic effect), or alternatively, the resistance effect might not change indicating that all three genes act in the same pathway (neutral effect, a form of classic epistasis). By conducting the DNA damage assay with MMS in all deletion combinations, the phenotype was highly similar, with around 10-fold higher resistance in the different mutation combinations compared to the WT strain (Fig. 5). These results suggest that *cas3* most likely participates with *mre11-rad50*, by restraining HR and thereby allowing MMEJ to act as primary DSB repair pathway.

### *Cas3* decreases the efficiency of HR

To obtain more direct results about the role of *cas3* in HR pathway, we decided to execute a “pop-in” recombination assay (Dattani et al. 2022), which enabled us to measure the integration efficiency of a “suicide” plasmid (that does not contain a *H. volcanii* origin of replication), into the *Δcas3* mutant genome, which could be achieved only via HR of the plasmid with the chromosome.

**Figure 6.**
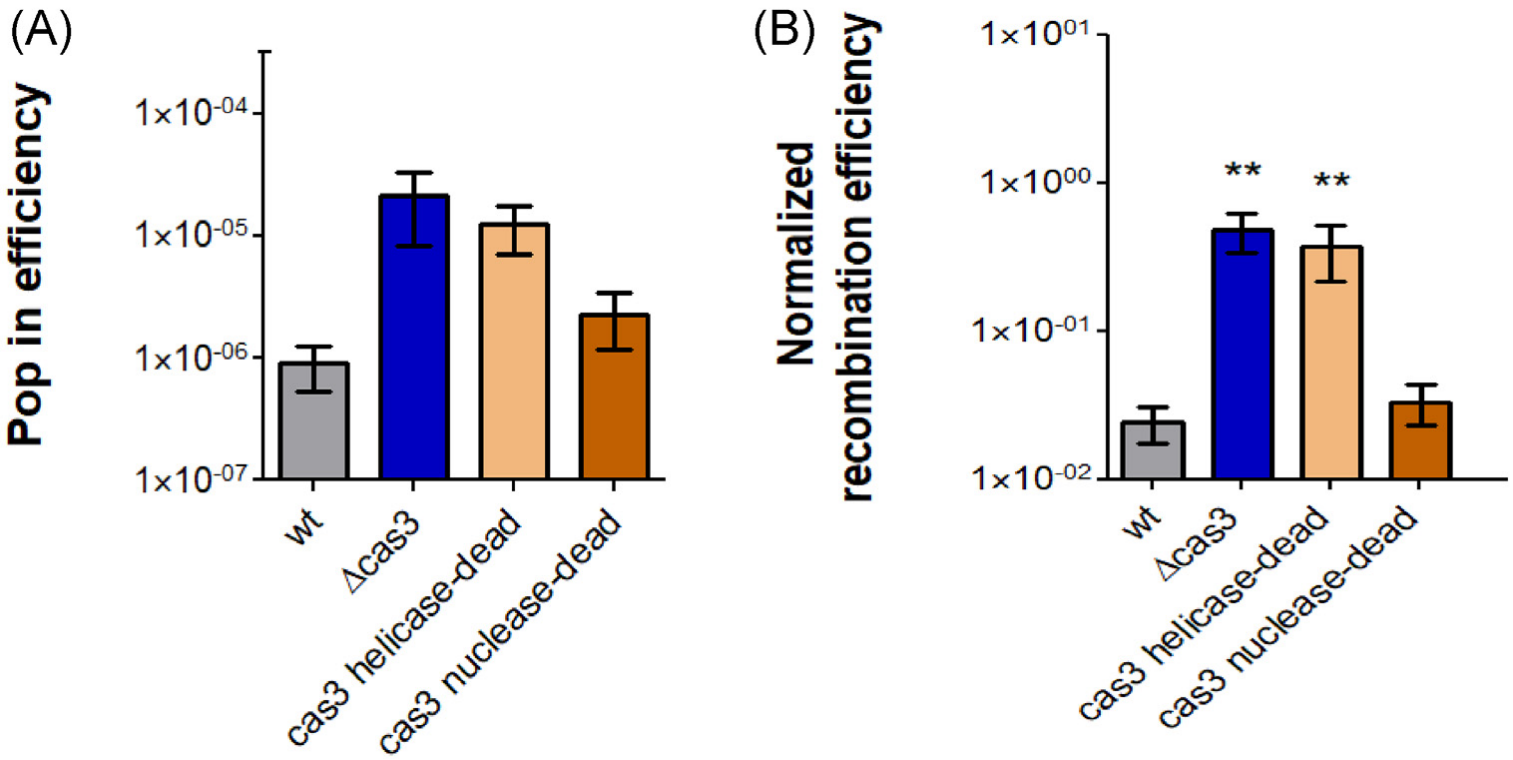
Pop-in / Recombination efficiencies of *cas3* mutants. Integrative plasmid (pTA131-*metX volcanii*) was transformed into WT (H26), *Δcas3* (UG610), *cas3 helicase-dead* (UG750), and *cas3 nuclease-dead* (UG767). (A) Pop-in transformation efficiency was calculated as the number of CFU produced by successfully transformed cells with the integrative plasmid divided by the total cells in at least four biological replicates. (B) Normalized recombination efficiency values were obtained by dividing the transformation efficiency obtained with the integrative plasmid (pTA131-*metX-volcanii*) divided by the transformation efficiency with a replicating plasmid (pTA927) in at least four biological replicates. ** = *P*-value <.01, Mann–Whitney test, compared to WT.

When transforming the *Δcas3* mutant with the integrative plasmid, higher pop-in (recombination) efficiency was obtained compared to WT (Fig. 6 A), suggesting an increased HR efficiency in the *Δcas3* mutant (see above). To validate that these differences are HR-based and not due to transformation efficacy, we performed a transformation efficiency assay with the replicative plasmid pTA927 (containing a *H. volcanii* origin of replication, which does not require recombination for successful transformation). The control assay resulted in a minor and insignificant differences in transformation efficiency between WT and Δ*cas3* strains (Supplementary Fig. S4). Normalized recombination efficiency values were obtained by dividing the pop-in transformation efficiency values (using the integrative plasmid) by the transformation efficiency values (obtained for the same strains using the replicating plasmid). This calculation showed the same trend, namely that the *Δcas3* mutant has higher HR rates than the WT by one order of magnitude (Fig. 6 B). Furthermore, when these transformation assays were performed in *cas3 nuclease-dead* and *cas3 helicase-dead* backgrounds, they revealed that deficiency in Cas3 helicase domain was not only responsible for greater cellular survival following MMS treatment (Fig. 4), but also for the increased rate of HR (Fig. 6B). A strain deleted in all *cas* genes showed an intermediate phenotype, closer to that of the WT (Supplementary Fig. S6).

## Discussion

CRISPR-Cas systems play important roles in prokaryotic immunity. The three stages of the CRISPR-Cas immunity process (adaptation, maturation, and interference) have been well studied and characterized, and the function of the *cas* genes has been elucidated in several types and subtypes, including I-B systems, such as that of the haloarchaeon *H. volcanii* (Maier et al. 2015). Recent work has shown that at least one protein, namely Cas1, also participates in DNA repair (Babu et al. 2011; Wörtz et al. 2022). However, the role of other *cas* genes in DNA repair is still unknown. Here we provide evidence for involvement of Cas3 in the process of DNA repair in archaea, potentially in restraining the HR pathway.

By conducting survival assays after UV exposure and MMS treatment, we have observed that a *H. volcanii Δcas3* mutant shows greater cell survival than the WT (Fig. 1, Supplementary Fig. S2). These results suggested a possible role for *cas3* gene in DNA damage response in *H. volcanii*. Surprisingly, although the *H. volcanii Δcas3* mutant was found to be more resistant to DNA damaging agents than the WT, its ability to quickly resume growth was impaired (Fig. 2). In addition, the RADAR-seq analysis revealed that its ability to quickly repair MMS lesions was also reduced. (Fig. 3). These observations suggest that WT and *Δcas3* cells utilize two different DNA repair strategies. While *Δcas3* uses a slower but more accurate repair strategy, this will benefit the organism only in noncompetitive situations, the WT uses a repair strategy with a decisive advantage in natural competition scenarios, because cells will be faster to resume growth and replication.

Delmas et al., (2009) previously showed that *H. volcanii mre11*-*rad50* mutant cells are more resistant to DNA damaging agents but are also slower to recover. Further, they demonstrated that the DNA repair strategy used in *mre11*-*rad50* mutants cells involves unrestrained HR, while in WT cells, DSBs were mainly repaired by MMEJ. By conducting DNA damage assays using MMS in all deletion combinations (*mre11,rad50*, and*cas3*), we observed that the resistance phenotype did not show additivity. All combinations of deletions showed an about 10-fold higher resistance on average, compared with the WT strain (Fig. 5). Furthermore, similar to deletion of the *mre11-rad50* genes, deletion of *cas3* also leads to increased efficiency in HR (Fig. 6 B). These results suggest that Cas3 participates with Mre11-Rad50 in restraining HR, thereby allowing the faster MMEJ to act as primary DSB repair pathway. It is believed that restraining HR may hold a significant advantage for a highly polyploid organism, such as *H. volcanii*, leading to a to ensure rapid repair of DSBs at the expense of accuracy by using MMEJ in the first step, and later using HR to resolve any mutations and thereby maintain genome fidelity in the second step (Delmas et al. 2009).

Analysis of *cas3* mutations revealed that the helicase activity of Cas3 is responsible for both DNA damage sensitivity (Fig. 4) and decreased HR efficiency (Fig. 6 B). These results provide a starting point for a mechanistic understanding that needs to be further explored. Our (nonexclusive) hypothesis, which best fits with the results, is that Cas3 helicase activity functions in restraining HR, possibly by removal of the D-loops and/or recombinase proteins to prevent HR. Consequently, this activity of Cas3 promotes the alternative MMEJ mechanism to act as the primary DSB repair pathway.

CRISPR-Cas systems require DNA nucleases and helicases to exert protection from selfish elements, and therefore are prone to interactions with the DNA repair machinery enzymes that perform these functions. Bernheim et al. (2019) suggested that the repertoire of DNA DSB repair systems an organism possesses determines to a great extent, which CRISPR-Cas systems it can acquire, if any, and showed that different subtypes are associated (either negatively or positively) with different repair components. It has been observed, e.g. that type II-A CRISPR-Cas systems in bacteria are very rarely found in genomes that also have the machinery for nonhomologous end joining that requires the Ku protein, probably because the Cas protein Csn2 interferes with this mechanism of DNA repair (Bernheim et al. 2017).

AddAB, which is involved in strand resection and in some ways is functionally analogous to Mre11-Rad50, is negatively associated with the I-B subtype in Firmicutes, but positively associated with this subtype in Proteobacteria (Bernheim et al. 2019). This implies that interactions between archaeal I-B systems and DNA repair are not just possible but rather probable. Indeed, recent work in *H. volcanii* has shown that the Cas1 integrase, whose primarily role is to integrate new spacers into the CRISPR array, is important for cellular survival of DNA damage caused by oxidative stress and that it can substitute for the DNA repair enzyme Flap endonuclease 1 (Fen1) in cleaving branched intermediate structures (flaps) (Wörtz et al. 2022). Given that such flap structures are also formed during MMEJ and our observations that Cas3 is involved in the MMEJ process, it is likely that both Cas1 and Cas3 can independently be found at repair sites. This could help direct spacer acquisition to elements already cut by other defensive nucleases such as restriction enzymes, but also cause acquisition of self-targeting “spacers” (Stachler et al. 2017, 2020). An additional advantage may be that by restraining HR, Cas3 will reduce the chances of foreign DNA elements, such as plasmids [which the *H. volcanii* system successfully targets and destroys (Fischer et al. 2012)], recombining with the host chromosome and integrated into it via shared insertion sequences or other repetitive sequences present in both the DNA of both the invader and the host.

Our results imply a certain degree of coevolution between CRISPR-Cas systems and the core DNA repair machinery, which is surprising given that CRISPR-Cas systems in halophilic archaea tend to be plasmid-encoded and have been frequently transferred by horizontal gene transfer, and that some *Haloferax* strains, such as *H. gibonsii* LR2-5 do not encode CRISPR-Cas systems (Mizuno et al. 2019; Tittes et al. 2021). It will be interesting to compare the DNA damage response of such naturally CRISPR-deficient strains to their CRISPR-positive relatives, which will help address the question of under which conditions, if any is the net contribution of CRISPR-Cas to DNA repair positive rather than negative and whether it can select in favor of CRISPR-Cas retention even under low viral pressures. However, since having an intact CRISPR-Cas reduces the frequency of HR (Fig. 6), it can reduce the rates of horizontal gene transfer in this and other species of halophilic archaea (Naor et al. 2012), and thus have undesirable evolutionary consequences.

## Materials and methods

### Strains, plasmids, and oligonucleotides

Strains are shown in Table S1, plasmids in Table S2, and oligonucleotides in Table S3.

### Culture conditions

*H. volcanii* strains were routinely grown aerobically at 45°C in either Hv-YPC (rich medium) or in Hv-Ca / Hv-Ca^+^ (minimal medium) as described in (Allers et al. 2004). Hv-YPC containing (per liter) 144 g of NaCl, 21 g of MgSO_4_∙7H_2_O, 18 g of MgCl_2_∙6H_2_O, 4.2 g of KCl, and 12 mM Tris HCl (pH 7.5). For solid media, agar (Difco) was added at a concentration of 15 g per liter. 0.5% (w/v) yeast extract (Difco), 0.1% (w/v) peptone (Oxoid), and 0.1% (w/v) casamino acids (Difco) were added, and the medium was autoclaved. After cooling, CaCl_2_ was added to a final concentration of 3 mM. Casamino acids medium (Hv-Ca) was made in a similar manner, except that yeast extract and peptone were excluded, casamino acids were added to a final concentration of 0.5% (w/v), 0.8 mg of thiamine, and 0.1 mg of biotin were added per liter. Enhanced Ca (Hv-Ca^+^) contained the same concentration of salts as Hv-Ca, except that Tris HCl (pH 7.5) was added to a concentration of 42 mM. After autoclaving and cooling, 4.25 ml of a sodium DL-lactate solution (60%, w/v), 3.83 g of disodium succinic acid ∙ 6H2O, 0.25 ml of glycerol, 5 ml of a 1 M NH_4_Cl solution, 6 ml of a 0.5 M CaCl_2_ solution, 2 ml of 0.5 M potassium phosphate buffer (pH 7.5), 1 ml of trace elements solution (Mevarech and Werczberger 1985), 0.8 mg of thiamine, and 0.1 mg of biotin were added per liter. When required, thymidine was added to a concentration of 40 µg/ml, and uracil was added at a concentration of 50 µg/ml. For p.*tnaA* promoter activation, tryptophan was added to a final concentration of 2 mM (Large et al. 2007; Allers 2010; Allers et al. 2010). For pop-out selection medium, 5-FOA was added to a concentration of 50 µg/ml.

*Escherichia coli* strains DH12S and ns2626 were grown aerobically at 37°C in LB medium, containing 200 µg/ml of ampicillin when required. The latter strain was used to prepare unmethylated plasmid DNA for efficient transformation of *H. volcanii* (Holmes, Nuttall, and Dyall-Smith 1991).

### Plasmid cloning

pUG334 was generated as follows. Upstream and downstream constructs to *cas3* gene were amplified from *H. volcanii* genomic DNA using IS225/6 (up) and IS227/8 (down) primers. The primers contained additional restriction sites for NotI (5′-up), BamHI (3′-down), and additional 20 reverse complement bases (3′-up, 5′-down). Constructs were fused by overlapping polymerase chain reaction (PCR), then the product was digested with restriction enzyme and ligated to pTA131 (digested with NotI and BamHI).

pUG427 was generated as follows. Upstream construct to HVO_CRISPR_2 locus and downstream construct to HVO_CRISPR_3 locus were amplified from *H. volcanii* genomic DNA using IS307/9 (up) and IS308/10 (down) primers. The primers contained additional restriction sites for BamHI (5′-up), HindIII (3′-down) and additional 19 reverse complement bases (3′-up, 5′-down). Constructs were fused by overlapping PCR, then the product was digested with restriction enzyme and ligated to pTA131 (digested with BamHI and HindIII).

pUG523 was generated as follows. Upstream and downstream constructs of *cas3* gene were amplified from *H. volcanii* genomic DNA using IS596/7 (up) and IS598/9 (down) primers. The primers contained additional 20 homologous bases to pTA131 cloning site (5′-up, 3′-down) and additional 15 reverse complement bases to each other (3′-up, 5′-down). pTA131 was amplified and linearized using IS600/1 primers. The purified products were cloned together through gibson assembly.

pUG536 was generated as follows. Upstream and downstream constructs of *cas4* gene were amplified from *H. volcanii* genomic DNA using IS590/1 (up) and IS592/3 (down) primers. The primers contained additional 20 homologous bases to pTA131 cloning site (5′-up, 3′-down) and additional 15 reverse complement bases to each other (3′-up, 5′-down). pTA131 was amplified and linearized using IS594/5 primers. The purified products were cloned together through gibson assembly.

pUG636 was generated by GeneScript. Synthetic fragment of the *cas3 H. volcanii* gene contained the HD63-64AA double mutation flanked by NdeI - ATG - 3x flag from the 5′ terminus and TGA - NotI from the 3′ terminus cloned into pTA927 using restriction enzymes and ligation.

pUG734 was generated as follows. *Cas3* helicase dead gene was amplified from pTA927-cas3-D444A-Flag-N using GM23/4 primers. The primers contained additional restriction sites for BamHI (5′) and EcoRI (3′). Product was digested with restriction enzyme and ligated to pTA131 (digested with BamHI and EcoRI).

pUG753 was generated as follows. Upstream construct of *cas3* gene was amplified from *H. volcanii* genomic DNA using GM29/30 (up) primers and downstream construct of *cas3* nuclease dead was amplified from pUG636 using GM31/2 (down) primers. The primers contained additional restriction sites for HindII (5′-up) and BamHI (3′-down). Constructs were fused by overlapping PCR, then the product was digested with restriction enzyme and ligated to pTA131 (digested with HindIII and BamHI).

### Construction of *H. volcanii* strains

Strain construction was performed according to the pop-in/pop-out protocol described in (Bitan-Banin, Ortenberg, and Mevarech 2003; Allers et al. 2004). In this method, the upstream and downstream flanking regions of the sequence to be exchanged are amplified by PCR and cloned together into the “suicide plasmid” pTA131, that cannot replicate autonomously and carries the *pyrE*2 selectable genetic marker. The plasmids are then transformed into *H. volcanii ΔpyrE2* mutants, and the transformants, in which the plasmids have been integrated into the chromosome, are selected for on plates that lack uracil (“pop-in”). Upon counter-selection on plates containing uracil and 5-FOA, the only cells that survive are those in which the integrated plasmids have been excised by spontaneous intrachromosomal homologues recombination (“pop-out”), either restoring the WT gene or resulting in allele exchange. The “pop-out” strains were screened using pairs of primers located upstream and downstream to the desired deletion site and the sequence validated by Sanger sequencing. *Δcas genes,cas3-KO,Δcas3,Δcas4,cas3*helicase dead, and*cas3 nuclease dead* were constructed using the pop-in pop-out methodology as described above. Parental strains and plasmids used for the constructions are listed in Table S1 and S2.

### Transformation

Transformation of *H. volcanii* was carried out using the polyethylene glycol 600 method as described in (Cline et al. 1989; Allers et al. 2004). A volume of 1.5 ml liquid culture were grown in Hv-YPC to OD_600nm_ of 1.5, and then centrifuged at 3 600 × *g* for 5 min. The supernatant was discarded, and the cells were resuspended in 200 μl spheroplasting solution (1 M NaCl, 27 mM KCl, 50 mM Tris HCl PH 8.5, and 15% sucrose) and incubated at room temperature for 5 min. A volume of 20 μl of 0.5 M EDTA were added and cells were incubated at room temperature for 10 min. A volume of 10 μl of purified plasmid DNA were mixed with 15 μl spheroplasting solution and 5 μl of 0.5 M EDTA, and added to the cells, followed by incubation of 5 min at room temperature. Subsequently, 250 μl of PEG solution (60% PEG 600 in spheroplasting solution) was added, and cells were incubated for 30 more minutes at room temperature. Following the incubation, 1 ml of regeneration solution (3.4 M NaCl, 175 mM MgSO_4_, 34 mM KCl, 5 mM CaCl_2_, 50 mM Tris HCl pH 7.5, and 15% sucrose) was added and cells were centrifuged at 6 000 rpm for 7 min. The supernatant was discarded, and cells were resuspended in Hv-YPC medium supplemented with 15% sucrose and left to incubate without shaking overnight at 37°C. The cultures were then transferred to a 37°C shaker and left for an incubation of three more hours, then washed and plated on selective media.

Transformation of *E. coli* was carried out by using a standard electroporation protocol (Sambrook, J. and Russell 2001).

### DNA damage assays

For UV radiation assays, cultures were grown in Hv-YPC broth to a mid-log phase (OD_600nm_ of 0.7), serially diluted (10-fold) in 18% saltwater and 20 µl aliquots spotted on Hv-YPC plates. After drying, plates were exposed to UV-C radiation (50–250 J m^−2^) in a Hoefer^TM^ UVC 500 ultraviolet crosslinker. Control plates were not exposed to UV radiation. Plates were then covered by aluminum foil to prevent the penetration of visible light.

For chemical mutagenesis assays, mid-log phase cultures were divided into 2 ml aliquots and methyl methanesulphonate (MMS) was added (0.1–0.7 mg/ml final concentration). Cultures were returned to 45°C for 1 h, serially diluted (10-fold) and 20 µl aliquots were spotted on Hv-YPC plates.

In both UV and MMS exposure experiments, survivor colonies were counted after 5–10 days of incubation. Survival rate was calculated by dividing the number of colonies grown on the irradiated plates by the number of colonies grown on the control plates.

For the post-MMS recovery experiments, the MMS was removed after the first hour by centrifugation followed by resuspension in an equal volume of fresh Hv-YPC medium (X2), and the cultures were left to recover in 45°C incubation for different time points.

### Growth curves

To compare the growth of the *H. volcanii* strains and mutants, each sample was grown over-night in appropriate media at 45°C to the mid-log phase and then diluted to a fresh medium to OD_600nm_ of 0.1. The growth curves were carried out in 96-well plates at 45°C with continuous shaking, using the Biotek ELX808IU-PC microplate reader. Optical density was measured every 30 min at a wavelength of 595 nm.

### DNA extraction

Total genomic DNA extraction of *H. volcanii* was done by DNA spooling protocol described in (Allers et al. 2004). Saturated culture grown in Hv-YPC broth, with or without MMS, was centrifuged at 6 000 rpm for 5 min and resuspended in 200 μl of ST buffer (1 M NaCl, 20 mM Tris HCl pH 7.5). Then 200 μl of lysis solution (100 mM EDTA pH 8.0, 0.2% SDS) was added to lyse the cells. The aqueous phase was overlaid with 1 ml 70% ethanol and the DNA was spooled onto a glass capillary until liquid was homogeneous and clear. The DNA washed three times by transferring the spooled DNA to a microcentrifuge tube with 1 ml fresh 70% ethanol, and then allowed to dry. DNA was then solubilized in 100 μl TE (10 mM Tris HCl pH 7.5, 1 mM EDTA pH 8.0) containing 0.1 mg of RNase A per ml.

Plasmid extraction from *E. coli* was done by Sigma–Aldrich’s GenElute™ Plasmid Miniprep Kit.

### DNA damage quantification

Damaged DNA (by MMS) and undamaged DNA extractions were sequenced by RAre DAmage and Repair (RADAR) sequencing method (Zatopek et al. 2019). Briefly, PacBio libraries are created from isolated genomic DNA by shearing into 2 kb fragments and ligation of PacBio SMRTbell adapters. Nick translation is performed on PacBio libraries, in which hAAG (human alkyladenine DNA glycosylase), in combination with Endonuclease IV, nick the DNA backbone at 3 mA and 7 mG (caused by the MMS). *Bst* FL DNA polymerase, a dNTP pool containing dTTP, dGTP, d6mATP, and d4mCTP, Taq DNA ligase and NAD+, were used to replace the nick with a patch of modified bases. Nick translated libraries are sequenced using PacBio SMRT sequencing on Sequel instrument, followed by downstream analysis to determine location and frequency of patches per million bases sequenced. The amount of the total sequenced patches is equal to the amount of the DNA lesions present in the sequenced sample.

### Measuring transformation / recombination efficiencies

Transformation efficiencies were calculated for *H. volcanii* strains after transforming them with pTA927, a replicating plasmid or with pTA131+*metX-volcanii*, “suicide” integrative plasmid (both carrying the *pyrE2* marker for selection). Transformation efficiency was calculated as the number of successfully transformed CFUs with the replicating plasmid (pTA927) grown on the selective plates (Hv-Ca) divided by the number of CFU grown on nonselective plates (Hv-YPC). Pop-in transformation could be achieved only by a recombination event between the plasmid *metX* gene and the chromosomal *metX*, leading to integration of the plasmid into the *H. volcanii* chromosome. Pop-in transformation efficiency was calculated as the number of successfully transformed CFUs with the integrative plasmid (pTA131+*metX-volcanii*) grown on the selective plates (Hv-Ca) divided by the number of CFU grown on nonselective plates (Hv-YPC). Recombination efficiency values were obtained by dividing the transformation efficiency obtained with the integrative plasmid (pTA131-*metX-volcanii*) divided by the transformation efficiency with a replicating plasmid (pTA927).

## Supplementary Material

uqad007_Supplemental_File

## References

[bib1] Allers T . Overexpression and purification of halophilic proteins in *Haloferax Volcanii*. Bioeng Bugs. 2010;1:290–2. 10.4161/bbug.1.4.11794 PMC3026470 21327063

[bib2] Allers T , Barak S, Liddell S et al. Improved strains and plasmid vectors for conditional overexpression of His-tagged proteins in *Haloferax Volcanii*. Appl Environ Microbiol. 2010;76:1759–69. 20097827 10.1128/AEM.02670-09 PMC2838008

[bib3] Allers T , Ngo HP, Mevarech M et al. Development of additional selectable markers for the halophilic archaeon *Haloferax Volcanii*based on the leuB and trpA Genes. Appl Environ Microbiol. 2004;70:943–53. 14766575 10.1128/AEM.70.2.943-953.2004 PMC348920

[bib4] Babu M , Beloglazova N, Flick R et al. A dual function of the CRISPR-Cas system in bacterial antivirus immunity and DNA repair. Mol Microbiol. 2011;79:484–502.. doi: 10.1111/j.1365-2958.2010.07465.x. 21219465 10.1111/j.1365-2958.2010.07465.x PMC3071548

[bib5] Beloglazova N , Petit P, Flick R et al. Structure and activity of the Cas3 HD nuclease MJ0384, an effector enzyme of the CRISPR interference. EMBO J. 2011;30:4616–27.. doi: 10.1038/emboj.2011.377. 22009198 10.1038/emboj.2011.377 PMC3243599

[bib6] Beranek DT . Distribution of methyl and ethyl adducts following alkylation with monofunctional alkylating agents. Mutation Research/Fundamental and Molecular Mechanisms of Mutagenesis. 1990;231:11–30. 2195323 10.1016/0027-5107(90)90173-2

[bib7] Bernheim A , Bikard D, Touchon M et al. A matter of background: DNA repair pathways as a possible cause for the sparse distribution of CRISPR-Cas systems in bacteria. Philos Trans R Soc B Biol Sci. 20180088, 2019;374. 10.1098/rstb.2018.0088 PMC6452273 30905287

[bib8] Bernheim A , Calvo-Villamañán A, Basier C et al. Inhibition of NHEJ repair by Type II-A CRISPR-Cas systems in bacteria. Nat Commun. 2017;8(1):2094. 29234047 10.1038/s41467-017-02350-1 PMC5727150

[bib9] Bitan-Banin G , Ortenberg R, Mevarech M. Development of a gene knockout system for the halophilic archaeon *Haloferax Volcanii*by use of the *pyrE* gene. J Bacteriol. 2003;185:772–8. 12533452 10.1128/JB.185.3.772-778.2003 PMC142808

[bib10] Blackwood JK , Rzechorzek NJ, Bray SM et al. End-resection at DNA double-strand breaks in the three domains of life. Biochem Soc Trans. 2013;41:314–20.. doi: 10.1042/BST20120307. 23356304 10.1042/BST20120307 PMC3561678

[bib11] Brendel J , Stoll B, Lange SJ et al. A complex of Cas proteins 5, 6, and 7 is required for the biogenesis and stability of clustered regularly interspaced short palindromic repeats (CRISPR)-derived RNAs (CrRNAs) in *Haloferax Volcanii*. J Biol Chem. 2014;289:7164–77. 24459147 10.1074/jbc.M113.508184 PMC3945376

[bib12] Cline SW , Lam WL, Charlebois RL et al. Transformation methods for halophilic archaebacteria. Can J Microbiol. 1989;35:148–52.. doi: 10.1139/m89-022. 2497937 10.1139/m89-022

[bib13] Dattani A , Sharon I, Shtifman-Segal E et al. Differences in homologous recombination and maintenance of heteropolyploidy between *Haloferax volcanii* and *Haloferax mediterranei*, *G3 In press* 2022.https://doi.org/10.1101/2022.03.26.485934 10.1093/g3journal/jkac306 PMC10085750 36454095

[bib14] Delmas S , Duggin IG, Allers T. DNA damage induces nucleoid compaction via the Mre11-Rad50 complex in the archaeon *Haloferax Volcanii*. Mol Microbiol. 2013;87:168–79.. doi: 10.1111/mmi.12091. 23145964 10.1111/mmi.12091 PMC3565448

[bib15] Delmas S , Shunburne L, Ngo HP et al. Mre11-Rad50 promotes rapid repair of DNA damage in the polyploid archaeon *Haloferax Volcanii* by restraining homologous recombination. PLos Genet. 2009;5:e1000552 19593371 10.1371/journal.pgen.1000552 PMC2700283

[bib16] Fischer S , Maier LK, Stoll B et al. An archaeal immune system can detect multiple protospacer adjacent motifs (PAMs) to target invader DNA, J Biol Chem. 2012;287, 33351–63. 22767603 10.1074/jbc.M112.377002 PMC3460438

[bib19] Gong B , Shin M, Sun J et al. Molecular insights into DNA interference by CRISPR-associated nuclease-helicase Cas3. Proc Natl Acad Sci. 2014;111:16359–64.. doi: 10.1073/pnas.1410806111. 25368186 10.1073/pnas.1410806111 PMC4246338

[bib21] Haft DH , Selengut J, Mongodin EF et al. A guild of 45 CRISPR-associated (Cas) protein families and multiple CRISPR/Cas subtypes exist in prokaryotic genomes. PLoS Comput Biol. 2005;1:0474–83. 10.1371/journal.pcbi.0010060 PMC1282333 16292354

[bib22] Hille F , Richter H, Wong SP et al. The biology of CRISPR-Cas: backward and forward. Cell. 2018;172:1239–59.. doi: 10.1016/j.cell.2017.11.032. 29522745 10.1016/j.cell.2017.11.032

[bib23] Holmes ML , Nuttall SD, Dyall-Smith ML. Construction and use of halobacterial shuttle vectors and further studies on Haloferax DNA gyrase. J Bacteriol. 1991;173:3807–13. 1711028 10.1128/jb.173.12.3807-3813.1991 PMC208012

[bib24] Howard JAL , Delmas S, Ivančić-Baće I et al. Helicase dissociation and annealing of RNA-DNA hybrids by *Escherichia coli*Cas3 protein. Biochem J. 2011;439:85–95.. doi: 10.1042/BJ20110901. 21699496 10.1042/BJ20110901

[bib25] Large A , Stamme C, Lange C et al. Characterization of a tightly controlled promoter of the halophilic archaeon *Haloferax Volcanii*and its use in the analysis of the essential cct1 gene. Mol Microbiol. 2007;66:1092–106. 17973910 10.1111/j.1365-2958.2007.05980.x

[bib27] Lindahl T , Wood RD. Quality control by DNA repair. Science. 1999;286:1897–905.. doi: 10.1126/science.286.5446.1897. 10583946 10.1126/science.286.5446.1897

[bib28] Lundin C , North M, Erixon K et al. Methyl methanesulfonate (MMS) produces heat-labile DNA damage but no detectable in vivo DNA double-strand breaks. Nucleic Acids Res. 2005;33:3799–811.. doi: 10.1093/nar/gki681. 16009812 10.1093/nar/gki681 PMC1174933

[bib29] Maier L-K , Dyall-Smith M, Marchfelder A. The adaptive immune system of Haloferax Volcanii. Life. 2015;5:521–37.. doi: 10.3390/life5010521. 25692903 10.3390/life5010521 PMC4390866

[bib30] Makarova KS , Aravind L, Grishin NV et al. A DNA repair system specific for thermophilic archaea and bacteria predicted by genomic context analysis. Nucleic Acids Res. 2002;30:482–96. 11788711 10.1093/nar/30.2.482 PMC99818

[bib31] Makarova KS , Grishin NV, Shabalina SA et al. A putative RNA-interference-based immune system in prokaryotes: computational analysis of the predicted enzymatic machinery, functional analogies with eukaryotic RNAi, and hypothetical mechanisms of action. Biol Direct. 2006;1:1–26. 16545108 10.1186/1745-6150-1-7 PMC1462988

[bib33] Marshall CJ , Santangelo TJ. Archaeal DNA repair mechanisms. Biomolecules. 2020;10: 1472.doi: 10.3390/biom10111472. 33113933 10.3390/biom10111472 PMC7690668

[bib34] Mevarech M , Werczberger R. Genetic transfer in *Halobacterium Volcanii*. J Bacteriol. 1985;162:461–2. 3980444 10.1128/jb.162.1.461-462.1985 PMC219016

[bib35] Mizuno CM , Prajapati B, Lucas-Staat S et al. Novel haloarchaeal viruses from Lake Retba infecting *Haloferax*and *Halorubrum*Species. Environ Microbiol. 2019;21:2129–47.. doi: https://doi.org/10.1111/1462-2920.14604. 30920125 10.1111/1462-2920.14604

[bib36] Mulepati S , Bailey S. Structural and biochemical analysis of nuclease domain of clustered regularly interspaced short palindromic repeat (CRISPR)-associated protein 3 (Cas3). J Biol Chem. 2011;286:31896–903.. doi: 10.1074/jbc.M111.270017. 21775431 10.1074/jbc.M111.270017 PMC3173111

[bib37] Naor A , Lapierre P, Mevarech M et al. Low species barriers in halophilic archaea and the formation of recombinant hybrids. Curr Biol. 2012;22:1444–8.. doi: 10.1016/j.cub.2012.05.056. 22748314 10.1016/j.cub.2012.05.056

[bib38] van der Oost J , Jore MM, Westra ER et al. CRISPR-based adaptive and heritable immunity in prokaryotes. Trends Biochem Sci. 2009;34:401–7.. doi: 10.1016/j.tibs.2009.05.002. 19646880 10.1016/j.tibs.2009.05.002

[bib39] Pérez-Arnaiz P , Dattani A, Smith V et al. Haloferax Volcanii—a model archaeon for studying DNA replication and repair. Open Biol. 2020;10:200293. doi: 10.1098/rsob.200293. 33259746 10.1098/rsob.200293 PMC7776575

[bib40] Sambrook J , Russell DW. Molecular Cloning: A Laboratory Manual, 1, 3rd edn, New York:Cold Spring Harbor Laboratory Press, 2001.

[bib41] Sinkunas T , Gasiunas G, Fremaux C et al. Cas3 is a single-stranded DNA nuclease and ATP-dependent helicase in the CRISPR/Cas immune system. EMBO J. 2011;30:1335–42. 21343909 10.1038/emboj.2011.41 PMC3094125

[bib43] Stachler A-E , Wörtz J, Alkhnbashi OS et al. Adaptation induced by self-targeting in a type IB CRISPR-Cas system. J Biol Chem. 2020;295:13502–15.. doi: 10.1074/jbc.RA120.014030. 32723866 10.1074/jbc.RA120.014030 PMC7521656

[bib44] Stachler AE , Turgeman-Grott I, Shtifman-Segal E et al. High tolerance to self-targeting of the genome by the endogenous CRISPR-Cas system in an archaeon. Nucleic Acids Res. 2017;45:5208–16. 28334774 10.1093/nar/gkx150 PMC5435918

[bib45] Tittes C , Schwarzer S, Pfeiffer F et al. Cellular and genomic properties of *Haloferax Gibbonsii*LR2-5, the host of euryarchaeal virus HFTV1. Front Microbiol. 2021;12:625599. doi: 10.3389/fmicb.2021.625599. 33664716 10.3389/fmicb.2021.625599 PMC7921747

[bib46] Westra ER , Erp PBG, Künne T et al. CRISPR immunity relies on the consecutive binding and degradation of negatively supercoiled invader DNA by Cascade and Cas3. Mol Cell. 2012;46:595–605. 22521689 10.1016/j.molcel.2012.03.018 PMC3372689

[bib47] White MF , Allers T. DNA repair in the archaea-an emerging picture. FEMS Microbiol Rev. 2018;42:514–26.. doi: 10.1093/femsre/fuy020. 29741625 10.1093/femsre/fuy020

[bib48] Wörtz J , Smith V, Fallmann J et al. Cas1 and Fen1 display equivalent functions during archaeal DNA repair. Front Microbiol. 2022;13:822304. doi: 10.3389/fmicb.2022.822304. 35495653 10.3389/fmicb.2022.822304 PMC9051519

[bib49] Zatopek KM , Potapov V, Maduzia LL et al. RADAR-Seq: a RAre DAmage and Repair sequencing method for detecting DNA damage on a genome-wide scale. DNA Repair (Amst). 2019;80:36–44.. doi: 10.1016/j.dnarep.2019.06.007. 31247470 10.1016/j.dnarep.2019.06.007

