# Supplementary material for: An archaeal Cas3 protein facilitates rapid recovery from DNA damage
Source: Microlife. 2023 Feb 9;4:uqad007. doi: 10.1093/femsml/uqad007 (PMC10117719; doi:10.1093/femsml/uqad007)
Supplement: uqad007_Supplemental_File [file uqad007_supplemental_file.docx]

**Supplementary information**

**Table S1: Strains used in this study.**

| strain | Genotype | Source^a^ / Reference |
| --- | --- | --- |
| *E. coli* | | |
| DH12S | *mcrA Δ(mrr-hsdRMS-mcrBC) Φ80d lacZΔM15 ΔlacX74 recA1 deoR Δ(ara, leu)7697 araD139 galU galK rpsL F'[proAB^+^ lacI^q^ZΔM15]* | Invitrogen |
| GM2198  (ns2626) | F^-^ *thr-1 araC14 leuB(Am) fhuA31 lacY1 tsx-78 glnX44(AS) galK2(Oc) λ^-^ dcm-6 hisG4(Oc) rpsL136 dam-13::Tn9 xylA5 mtl-1 thiE1* | (Marinus et al. 1983) |
| *H. volcanii* | | |
| H98 | ΔpHV2*, ΔpyrE2, ΔhdrB* | (Allers et al. 2004) |
| UG444 | ΔpHV2, *ΔpyrE2, ΔhdrB,* ΔHVO_pHV4: 204,805-218,601 | H98^b^ pUG427^c^ |
| UG356 | ΔpHV2*, ΔpyrE2, ΔhdrB, Δcas3:*212,781-215,615 | H98^b^ pUG334^c^ |
| H26 | ΔpHV2*, ΔpyrE2* | (Allers et al. 2004) |
| UG610 | ΔpHV2*, ΔpyrE2, Δcas3*:213,632-214,258 | H26^b^ pUG523^c^ |
| UG611 | ΔpHV2*, ΔpyrE2, Δcas4*:215,771-215,998 | H26^b^ pUG536^c^ |
| UG617 | ΔpHV2*, ΔpyrE2, Δcas3*::[*pyrE2^+^*, *cas3^+^*] | UG610^b^ pTA927-cas3-Flag-N^d^ |
| UG618 | ΔpHV2*, ΔpyrE2, Δcas3*::[*pyrE2^+^*, *cas3 H.D* (D444A)] | UG610^b^ pTA927-cas3-D444A-Flag-N^d^ |
| UG645 | ΔpHV2*, ΔpyrE2, Δcas3*::[ *pyrE2^+^*, *cas3 N.D* (HD63-64AA)] | UG610^b^ pUG636^d^ |
| H115 | ΔpHV2*, ΔpyrE2*, bgHa-Kp | (Delmas et al. 2009) |
| H202 | ΔpHV2*, ΔpyrE2*, bgHa-Kp, *Δrad50* | (Delmas et al. 2009) |
| H203 | ΔpHV2*, ΔpyrE2*, bgHa-Kp, *Δmre11* | (Delmas et al. 2009) |
| H204 | ΔpHV2*, ΔpyrE2*, bgHa-Kp, *Δmre11*, *Δrad50* | (Delmas et al. 2009) |
| UG669 | ΔpHV2*, ΔpyrE2*, bgHa-Kp, *Δmre11*, *Δcas3* | H203^b^ pUG523^c^ |
| UG670 | ΔpHV2*, ΔpyrE2*, bgHa-Kp, *Δrad50*, *Δcas3* | H202^b^ pUG523^c^ |
| UG671 | ΔpHV2*, ΔpyrE2*, bgHa-Kp, *Δmre11*, *Δrad50*, *Δcas3* | H204^b^ pUG523^c^ |
| UG750 | ΔpHV2*, ΔpyrE2, cas3* helicase-dead (D444A) | UG610^b^ pUG734^c^ |
| UG767 | ΔpHV2*, ΔpyrE2, cas3* nuclease-dead (HD63-64AA) | UG610^b^ pUG753^c^ |
| *Δcas* | ΔpHV2*, ΔpyrE2,* *Δcas (all cas genes deleted)* | H26^b^ |

^a^ Unless indicates otherwise, source of strains was this study.

^b^ Parental strain.

^c^ Pop in plasmid used for strain generation.

^d^ Replicative expression vector.

**Table S2: Plasmids used in this study.**

| Plasmid | Description | Source / Reference |
| --- | --- | --- |
| pTA131 | pBluescript II containing the *H. volcanii pyrE2* gene. | (Allers et al. 2004) |
| pUG123 | pTA131 with 1,115 bp *H. volcanii metX gene*. Inserted with HindIII and XbaI restriction sites - used for pop in efficiency assay. | (Naor et al. 2012) |
| pUG334 | pTA131 with 2,833 bp *Δcas3* construct. Inserted with NotI and BamHI restriction sites - used for the deletion of the complete *cas3* gene. | This study |
| pUG427 | pTA131 with 13,795 bp *Δcas genes* construct. Inserted with BamHI and HindIII restriction sites - used for the *cas* genes and both CRISPR loci deletion from pHV4. | This study |
| pUG523 | pTA131 with 627 bp *Δcas3* construct. Inserted through gibson assembly - used for *cas3* partial deletion. | This study |
| pUG536 | pTA131 with 228 bp *Δcas4* construct. Inserted through gibson assembly - used for *cas4* partial deletion. | This study |
| pUG734 | pTA131 with 2,648 bp *H. volcanii* *cas3* helicase dead gene (D444A). Inserted with BamHI and EcoRI restriction sites | This study |
| pUG753 | pTA131 with 2,927 bp *H. volcanii* *cas3* nuclease dead gene (HD63-64AA). Inserted with HindIII and BamHI restriction sites. | This study |
| pTA927 | Expression vector with *pyrE2* marker and pHV2 origin. | (Allers et al. 2010) |
| pTA927-cas3-Flag-N | pTA927 with *H. volcanii cas3* gene fused to N-terminal FLAG tag. | (Stachler and Marchfelder 2016) |
| pTA927-cas3-D444A-Flag-N | pTA927 with *H. volcanii cas3* helicase dead gene (D444A) fused to N-terminal FLAG tag. | (Stachler and Marchfelder 2016) |
| pUG636 | pTA927 with *H. volcanii cas3* nuclease dead gene (HD63-64AA) fused to N-terminal FLAG tag. | This study |

**Table S3: Oligonucleotides used in this study.**

| Primer | sequence (5’-3’) | properties |
| --- | --- | --- |
| IS225 | AAAAGCGGCCGCCGCGCTCAAGAATCAGACGA | *Δcas3* upstream forward primer, NotI site is underline, for generating *Cas3-KO.* |
| IS226 | CCCCCACCAACAGTAATCGCTTCGGTTAGCGGAAGACGAC | *Δcas3* upstream reverse primer, homologous sequence to downstream construct in red, for generating *Cas3-KO.* |
| IS227 | GCGATTACTGTTGGTGGGGG | *Δcas3* downstream forward primer, homologous sequence to upstream construct in red, for generating *Cas3-KO.* |
| IS228 | AAAAGGATCCAACTCTCGGAGCGTCTCAAC | *Δcas3* downstream reverse primer, BamHI site is underline, for generating *Cas3-KO.* |
| IS307 | AAAAGGATCCCTCGAAAACCGGCGCGTG | *Δcas genes* upstream forward primer, BamHI site is underline. |
| IS309 | GTTGTGGTGCGTCTTCGGC | *Δcas genes* upstream reverse primer, homologous sequence to downstream construct in red. |
| IS310 | GCCGAAGACGCACCACAACCAGACTCGGGCGCTACG | *Δcas genes* downstream forward primer, homologous sequence to upstream construct in red. |
| IS308 | AAAAAAGCTTAAGGGCGAGGGACTCGAAC | *Δcas genes* downstream reverse primer, HindIII site is underline. |
| IS590 | ACTGGCCGTCGTTTTACAACCGTAGACGCGATACTTCGCA | *Δcas4* upstream forward primer, homologous sequence to pTA131 in red, for *cas4* partial deletion. |
| IS591 | GGACGTCCCATAGCTGACATGACTGATGTTATCACGT | *Δcas4* upstream reverse primer, homologous sequence to downstream construct in red, for *cas4* partial deletion. |
| IS592 | AACATCAGTCATGTCAGCTATGGGACGTCCCGGCG | *Δcas4* downstream forward primer, homologous sequence to upstream construct in red, for *cas4* partial deletion. |
| IS593 | CAAGCGCGCAATTAACCCTCTGCCGTTTTTCGAATCGCAG | *Δcas*4 downstream reverse primer, homologous sequence to pTA131 in red, for *cas4* partial deletion. |
| IS594 | CTGCGATTCGAAAAACGGCAGAGGGTTAATTGCGCGCTTG | pTA131 forward primer, homologous sequence to downstream *Δcas*4 construct in red, for *cas4* partial deletion. |
| IS595 | TGCGAAGTATCGCGTCTACGACTGGCCGTCGTTTTACAAC | pTA131 reverse primer, homologous sequence to upstream *Δcas*4 construct in red, for *cas4* partial deletion. |
| IS596 | ACTGGCCGTCGTTTTACAACTGACTACGTCTACGCACAGC | *Δcas3* upstream forward primer, homologous sequence to pTA131 in red, for *cas3* partial deletion. |
| IS597 | GAGCGACGTGGTTTCTCGCTCGTCATTCAATGCCC | *Δcas3* upstream reverse primer, homologous sequence to downstream construct in red, for *cas3* partial deletion. |
| IS598 | TTGAATGACGAGCGAGAAACCACGTCGCTCCTCGA | *Δcas3* downstream forward primer, homologous sequence to upstream construct in red, for *cas3* partial deletion. |
| IS599 | CAAGCGCGCAATTAACCCTCCTCATCCTCGAACTGTGCCA | *Δcas3* downstream reverse primer, homologous sequence to pTA131 in red, for *cas3* partial deletion. |
| IS600 | TGGCACAGTTCGAGGATGAGGAGGGTTAATTGCGCGCTTG | pTA131 forward primer, homologous sequence to downstream *Δcas3* construct in red, for *cas3* partial deletion. |
| IS601 | GCTGTGCGTAGACGTAGTCAACTGGCCGTCGTTTTACAAC | pTA131 reverse primer, homologous sequence to upstream *Δcas3* construct in red, for *cas3* partial deletion. |
| GM23 | GCGGATCCATGACGTACCCACTCATATC | *cas3* forward primer, BamHI site is underline, for generating *Cas3 helicase-dead* |
| GM24 | GCGAATTCTCAGAACGTTGTGAACCGCC | *cas3* reverse primer, EcoRI site is underline, for generating *Cas3 helicase-dead* |
| GM29 | GCAAGCTTCTGGAACTCGACATCGGTGA | *cas3 N.D* upstream forward primer, HindIII site is underline, for generating *Cas3 nuclease-dead* |
| GM30 | GAGTGACGCCGCGACACAGA | *cas3 N.D* upstream reverse primer, for generating *Cas3 nuclease-dead* |
| GM31 | GAAGCGAACGACGGCGAGCG | *cas3 N.D* downstream forward primer, for generating *Cas3 nuclease-dead* |
| GM32 | GCGGATCCACGGCCGGTATCTCGAATTG | *cas3 N.D* downstream reverse primer, BamHI site is underline, for generating *Cas3 nuclease-dead* |


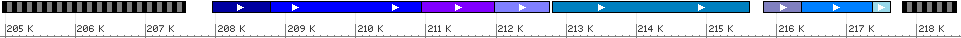


CRISPR array

cas6b

cas8b

cas7

cas5

cas3

cas1

cas2

cas4

CRISPR array

**cas3-KO**

**212,781 – 215,615**

**Δcas3**

**213,632 – 214,258**

**Δcas4**

**215,771 – 215,998**

**Δcas genes**

**204,805 – 218,601**

**Supplementary Figure 1. Construction of mutants.**

Map of the CRISPR locus and *cas* genes from pHV4 of *H. volcanii*. Locations of the deletions for *Δcas genes*, *cas3*-KO, *Δcas3,* and *Δcas4* mutants are shown.

**Supplementary Figure 2. *cas3*-KO mutant shows higher resistance to UV damage.**

WT (H98), *Δcas* (UG444) and *cas3*-KO (UG356) cultures were plated on Hv-YPC agar plates and exposed to UV. (A) Photographs were taken after 5-10 days (red arrows mark the resistant colonies). (B) Surviving colonies following different UV doses were enumerated, and the fraction of surviving cells in each strain was calculated. The mean and SE of at least three experiments are shown.

***Δcas* genes**

***cas3*-KO**

**wt**

10^0^

10^-1^

10^-2^

10^-3^

10^-4^

10^-5^

10^0^

10^-1^

10^-2^

10^-3^

10^-4^

10^-5^

**control**

**UV exposure (150 J/m^2^)**


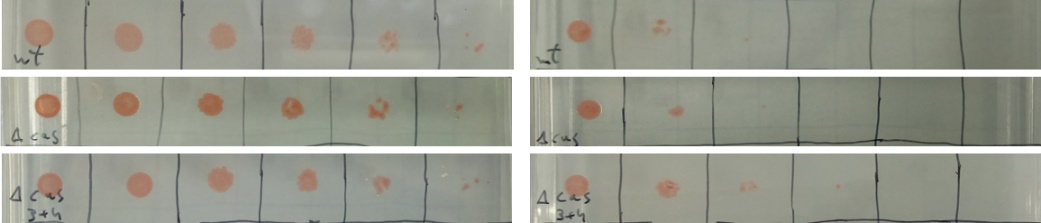

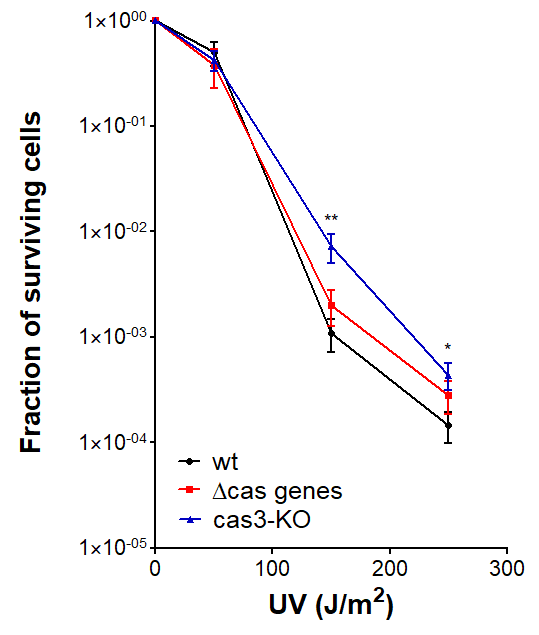


**A**

**B**

**Supplementary Figure 3. *Cas3* deletion does not impair growth rate.**

Growth curves of (A) WT (H26) and *Δcas3* (UG610) under optimal conditions are shown. The mean and SE of three biological replicates are shown for each time point. (B) Fraction of CFUs from either WT (H98) or *cas3-KO* (UG356) *H. volcanii* strains obtained in a direct (head to head) competition assay. Both strains were grown in liquid culture, aliquots were sampled, and the fraction of WT and mutant colony forming units was determined at different time points. Colors indicate independent biological replicates of the respective experiments.

**A**

**B**


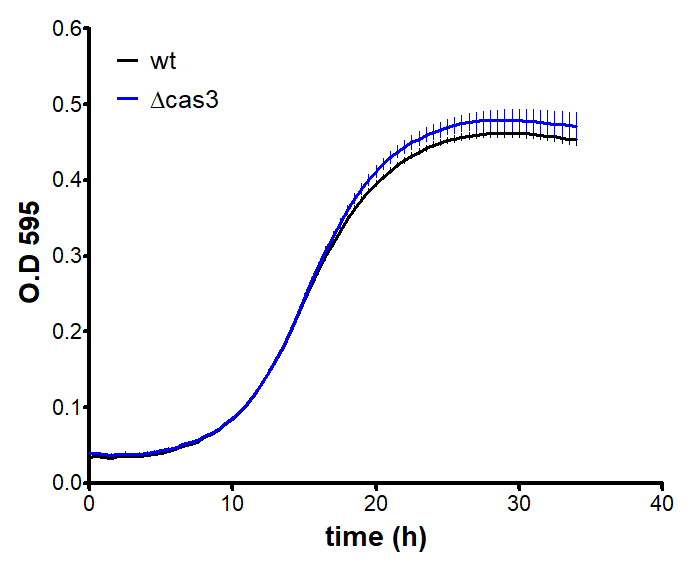

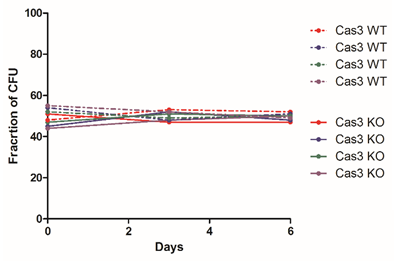

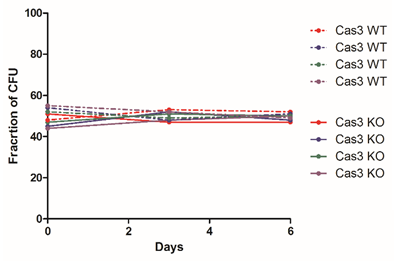


**Supplementary Figure 4. Transformation efficiencies of strains with different *cas3* genotypes.** A replicative plasmid (pTA927) was transformed into WT (H26), *Δcas3* (UG610), *cas3 helicase-dead* (UG750) and *cas3 nuclease-dead* (UG767). Transformation efficiency was calculated as the number of CFU obtained on selective divided by the total cells in at least four biological replicates.


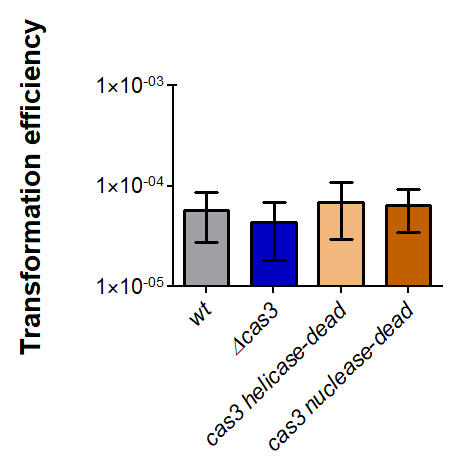

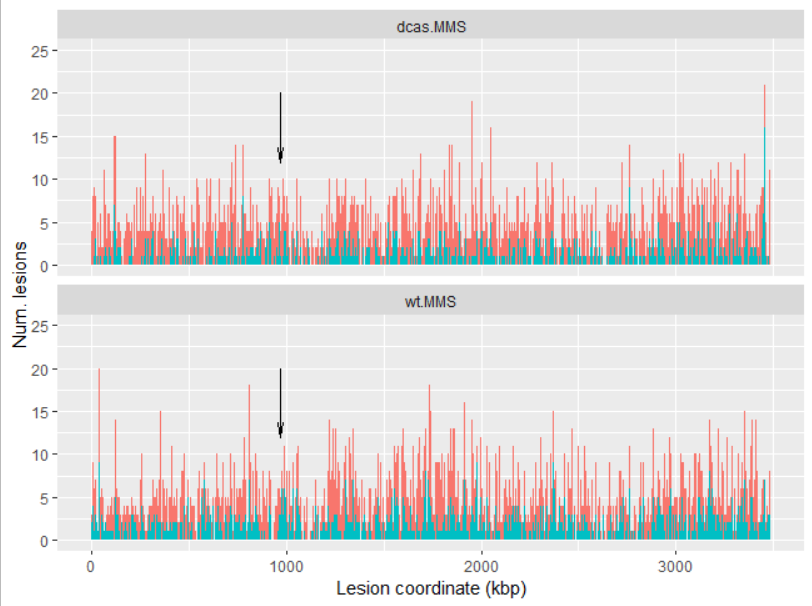


Integrated phv4

**Supplementary Figure S5. Lesions on the *H. volcanii* chromosome detected by RADAR-Seq are similar between cas3KO and w.t.**. Forward strand, bin size=348bp; the pHV4 plasmid is re-arranged and integrated at coordinate 249414 (schematic drawing), as is the case for many laboratory strains of *H. volcanii* (Hawkins et al., 2014). Location of the sequence that partially matches on of the *H. volcanii* spacers (Fischer et al., 2012) is indicated with an arrow. Blue and red lines each represent an independent biological replicate.


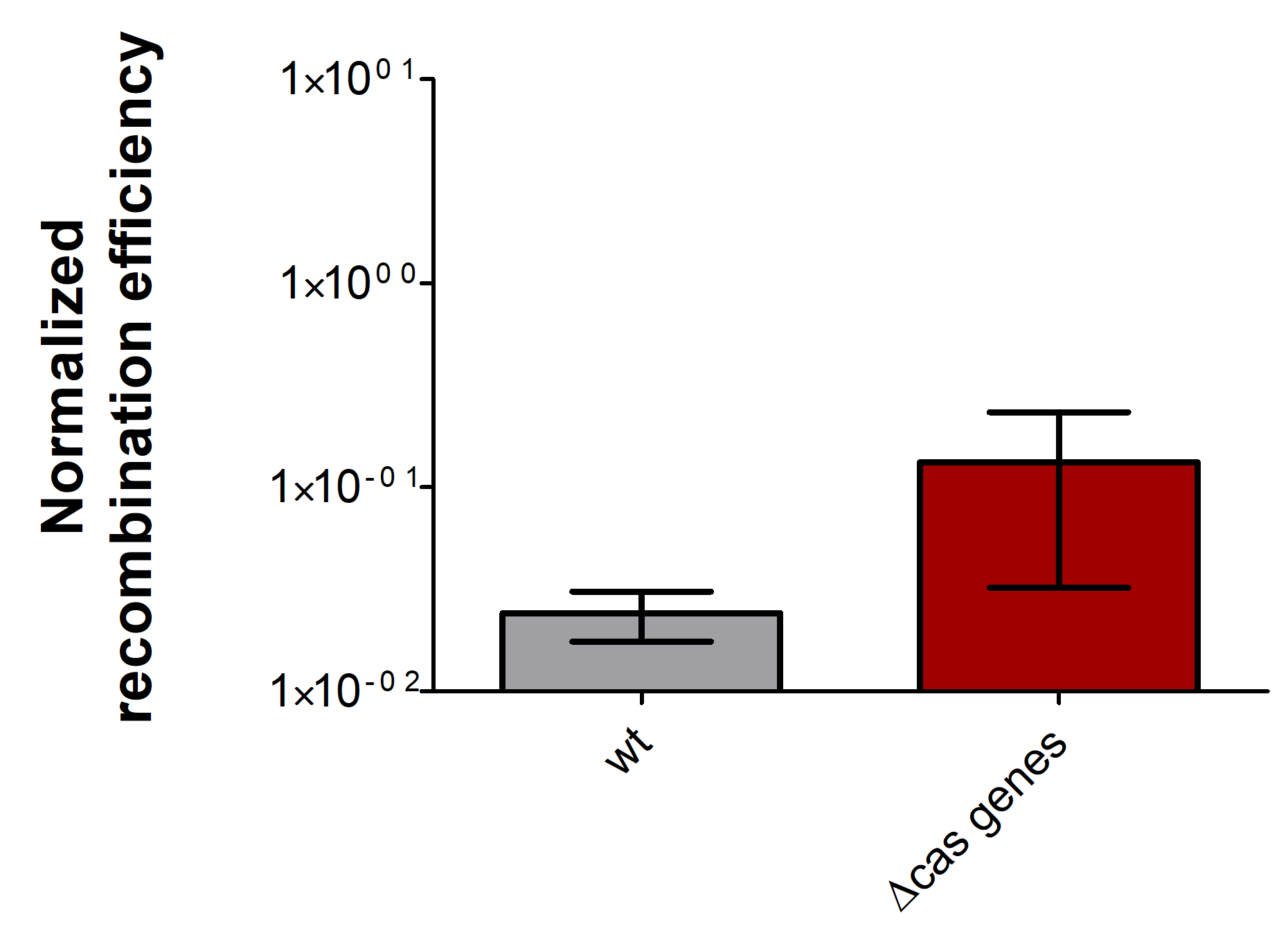


**Supplementary Figure 6. Pop-in** / **Recombination** **efficiencies of Δ*cas* mutants.**

Integrative plasmid (pTA131-*metX volcanii*) was transformed into WT (H26), and *Δcas.* Normalized recombination efficiency values were obtained by dividing the transformation efficiency obtained with the integrative plasmid (pTA131-*metX-volcanii*) divided by the transformation efficiency with a replicating plasmid (pTA927) in four biological replicates.
